# Stable overexpression and targeted gene deletion of the causative agent of ash dieback *Hymenoscyphus fraxineus*

**DOI:** 10.1186/s40694-023-00149-y

**Published:** 2023-01-13

**Authors:** Tobias Lutz, Birgit Hadeler, Mareike Jaeckel, Barbara Schulz, Cornelia Heinze

**Affiliations:** 1 Institute of Plant Science and Microbiology, Molecular Phytopathology, University of Hamburg, Ohnhorststr. 18, 22609 Hamburg, Germany; 2 Institute of Microbiology, Technische Universität Braunschweig, Spielmannstr. 7, 38106 Brunswick, Germany

**Keywords:** *Hymenoscyphus*, Ash dieback, Protoplast, Transformation, Homologous recombination

## Abstract

**Background:**

Due to the infection with the invasive ascomycete *Hymenoscyphus fraxineus*, which has been replacing the closely related and non-pathogenic native *Hymenoscyphus albidus*, the European ashes, *Fraxinus excelsior* (also known as the common ash), *Fraxinus angustifolia* (also known as narrow-leaved ash) and *Fraxinus ornus* (also known as the manna ash) are at risk. *Hymenoscyphus fraxineus* is the causative agent of ash dieback of the European ashes, but is non-pathogenic to the native Asian ash *Fraxinus mandshurica* (also known as the Manchurian ash). Even though the invasion of *H. fraxineus* is a great threat for ashes in Europe, the fungal biology is still poorly understood. By the use of live cell imaging and targeted gene knock-out, the fungal life cycle and host–pathogen interaction can be studied in more detail.

**Results:**

Here, we developed a protocol for the preparation of protoplasts from mycelium of *H. fraxineus*, for their regeneration and for stable transformation with reporter genes and targeted gene knock-out by homologous recombination. We obtained mutants with various levels of reporter gene expression which did not correlate with the number of integrations. In an in vitro infection assay, we demonstrated the suitability of reporter gene overexpression for fungal detection in plant tissue after inoculation. As a proof of principle for targeted gene knock-out, the hygromycin resistance cassette of a reporter gene-expressing mutant was replaced with a geneticin resistance cassette.

**Conclusions:**

The invasive fungal pathogen *H. fraxineus* is threatening the European ashes. To develop strategies for pest management, a better understanding of the fungal life cycle and its host interaction is crucial. Here, we provide a protocol for stable transformation of *H. fraxineus* to obtain fluorescence reporter strains and targeted gene knock-out mutants. This protocol will help future investigations on the biology of this pathogen.

**Supplementary Information:**

The online version contains supplementary material available at 10.1186/s40694-023-00149-y.

## Background

In Europe’s temperate zone, 3 ash species are naturally found: The common ash (*Fraxinus excelsior* L.), the manna ash (*Fraxinus ornus* L.) and the narrow-leaved ash (*Fraxinus angustifolia* Vahl). The common ash represents the most widely distributed species in Europe and provides high quality timber. This species is found as a pioneer but is also present in mature forests [1]. However, the tree population is at risk. The invasive fungal pathogen *Hymenoscyphus fraxineus* Baral, Queloz & Hosoya (syn. *Hymenoscyphus pseudoalbidus*) [2], which colonizes the native Asian Manchurian ash (*Fraxinus mandshurica* Rupr.) [3], made its first appearance in Europe in the 1990’s in Poland infecting the common ash [4]. This fungus is the causative agent of ash dieback of Europe’s ashes and is going to replace the closely related endemic *Hymenoscyphus albidus* (Roberge ex Gillet) W. Phillips 1887 [5, 6], which has been reported to be an infrequent and non-pathogenic species on European ashes [7]. While the common ash and the narrow-leaved ash are highly susceptible to *Hymenoscyphus fraxineus* (*H. fraxineus*), a lower susceptibility of the manna ash has been observed [8–10]. In contrast to the common ash, the Manchurian ash may be protected from ash dieback by inhibiting growth from the leaf to the shoot [11], since it was found that *H. fraxineus* is restricted to the leaves in this species [12].

Conidia are assumed to serve as spermatia for ascospore formation and not for spread [13]. Fones et al. [13] showed that mycelium, which germinated from the spermatia, was able to enter the vasculature and to form sporulating structures in the epidermis. Based on these results they concluded that these spermatia are potentially infectious.

Live cell imaging and targeted gene deletion are state-of-the-art techniques which are useful to obtain a more in-depth understanding of the fungal life cycle, host-fungus interaction and symptom development. For this, the stable expression of reporter genes and the possibility of targeted gene deletion are required. The complete genomic sequences of both, *H. fraxineus* and *H. albidus*, were published recently by Elfstrand et al. [14]. In combination with the protocol for stable transformation, which is provided here, targeted gene deletion will be possible in the future.

While higher plants often undergo *Agrobacterium tumefaciens*-mediated transformation or are transformed by the use of gene guns [15], many protocols for the genetic manipulation of fungi are based on protoplasts [16]. However, due to the variability of the fungal cell walls of different fungal species, enzymes for their degradation must be selected individually. Additionally, the osmotic pressure for integrity of the protoplasts is unique for each species [17]. The collection of a high number of protoplasts, their regeneration and the selection of an appropriate concentration of antibiotics for the transformed cells are further requirements, which are needed for the successful generation of stable transformants. In addition, the choice of appropriate promoters and terminators may be crucial for the overexpression of selection markers and the desired gene.

In our study, we provide a protoplast-based protocol for the transformation of *H. fraxineus* to obtain reporter strains and show their suitability for monitoring the fungal infection in situ. In addition, we deleted a specific gene by homologous recombination. Reporter strains and gene deletion mutants will help to study the biology of *H. fraxineus*. Further, this protocol enables viral transfection by either using particles or protoplast fusion as it was for instance reported by Kanematsu et al. [18] or Pingyan and Kaiying [19], respectively.

## Materials and methods

### Culture medium

Ash leaves (*Fraxinus excelsior* L.) were collected in late summer, washed in double distilled H_2_O (ddH_2_O) and stored at − 20 °C until use. Liquid ash medium (AM_L_, Table 1) was prepared by shredding 50 g of frozen ash leaves including petioles in 400 ml ddH_2_O. After autoclaving and incubation 16 h at room temperature (RT), the medium was filtered through 2 layers of cotton gauze and the filtrate was autoclaved again. Before use, floating particles were removed by centrifugation (20 min, 2000×*g*). The supernatant was adjusted to 1 L with sterile ddH_2_O. Solid ash medium (AM_S_, Table 1) was prepared with AM_L_ including 1.8% (w/v) microagar (Duchefa Biochemie, Haarlem, The Netherlands). For protoplasting, AM_L_ was additionally sterile filtered to remove particulate material (Millex-HA Syringe Filter Unit, 0.45 µm, mixed cellulose esters, 33 mm, ethylene oxide sterilized, Merck Millipore, Darmstadt, Germany).

**Table 1:** Media and buffers used in this study

Denomination	Description	Components
AM_L_	Liquid ash medium	50 g ash leaves incl. petioles in ddH_2_O^a^
AM_S_	Solid ash medium	AM_L_ 1.8% (w/v) microagar^a,b^
AM_RegL_ pH 5.8	Liquid ash medium for regeneration	AM_L_ 500 mM sucrose
AM_RegS_ pH 5.8	Solid ash medium for regeneration	AM_RegL_ 1.8% (w/v) microagar^b^
AM_HygS_	Solid ash medium with hygromycin	AM_S_ 100 µg/ml hygromycin B^b^
AM_GenS_	Solid ash medium with geneticin	AM_S_ 375 µg/ml geneticin disulphate (G418) solution^c^
Water agar		ddH_2_O 1.8% (w/v) microagar^b^
MgSO_4_-buffer pH 3.5/5.0/5.8	Cell wall digestion	1 M MgSO_4_ 50 mM tri-sodium citrate
STC pH 8.0	Collection of floating protoplasts	500 mM sucrose 10 mM Tris–HCl 50 mM CaCl_2_
PEG/STC pH 8.0	Transformation	STC 40% (w/v) PEG 4000
CTAB-buffer pH 8.0	gDNA isolation	2% (w/v) CTAB^d^ 100 mM Tris–HCl 20 mM EDTA 1.4 M NaCl
TE-buffer/RNase pH 8.0	gDNA isolation	10 mM Tris–HCl 1 mM EDTA 1 mg/ml RNaseA

^a^Details of preparation are given in the section “Culture medium”

^b^Duchefa Biochemie

^c^BioScience Grade, Carl Roth GmbH

^d^Cetyltrimethylammoniumbromid

### Enzyme solutions for protoplasting and restriction enzymes for DNA digestion

Driselase (1.75% w/v Driselase™ Basidiomycetes sp., Sigma-Aldrich, St. Louis, Missouri, USA), Lysing Enzymes (1.75% w/v, Lysing Enzymes from Trichoderma harzianum, Sigma-Aldrich) and chitinase (40 U/ml, ASA Spezialenzyme, Wolfenbüttel, Germany) were prepared in MgSO_4_-buffer (Table 1). For each set of experiments, the buffer was adjusted to pH 3.5, 5.0 or 5.8. The solutions were stirred for 30 min (200 rpm at RT), centrifuged for 10 min at 2000×*g* and the supernatant was sterile filtered (Millex-HA Syringe Filter Unit, 0.22 µm, mixed cellulose esters, 33 mm, ethylene oxide sterilized, Merck Millipore). For DNA restriction digestion, rCutSmart™ enzymes were used according to the manufacturers´ protocols (NEB, Ipswich, Massachusetts, USA).

### Fungal strain and culture conditions

The strain *H. fraxineus* NW-FVA 1856 was isolated from a stem necrosis of *F. excelsior*, collected in the Waldgehege Fahrenstedthof, mark 24860, Böklund, Abt. 3410a in Schleswig–Holstein, Germany, 2013 [20]. For propagation on solid medium, an agar block (ø 0.3 cm) was placed on an AM_S_ containing petri dish and incubated at RT in the dark. DNA extraction was performed from mycelium grown on AM_S_ covered with a cellophane sheet. For a liquid starter culture, 20 agar blocks (ø 0.3 cm) were excised from mycelium grown for 14 days on AM_S_ and transferred to a 100 ml Erlenmeyer flask containing 50 ml of AM_L_. After 14 to 28 days of static incubation at RT in the dark, the mycelium was rejuvenated. For that, the culture was blended twice for 10 s (Blender 800EBU, Waring, Torrington, Connecticut, USA) and diluted with an equal volume of AM_L_. Aliquots of 50 ml were statically incubated at RT in 100 ml Erlenmeyer flasks for 3 days for subsequent protoplasting.

### Constructs used for transformation

**Fig. 1:**
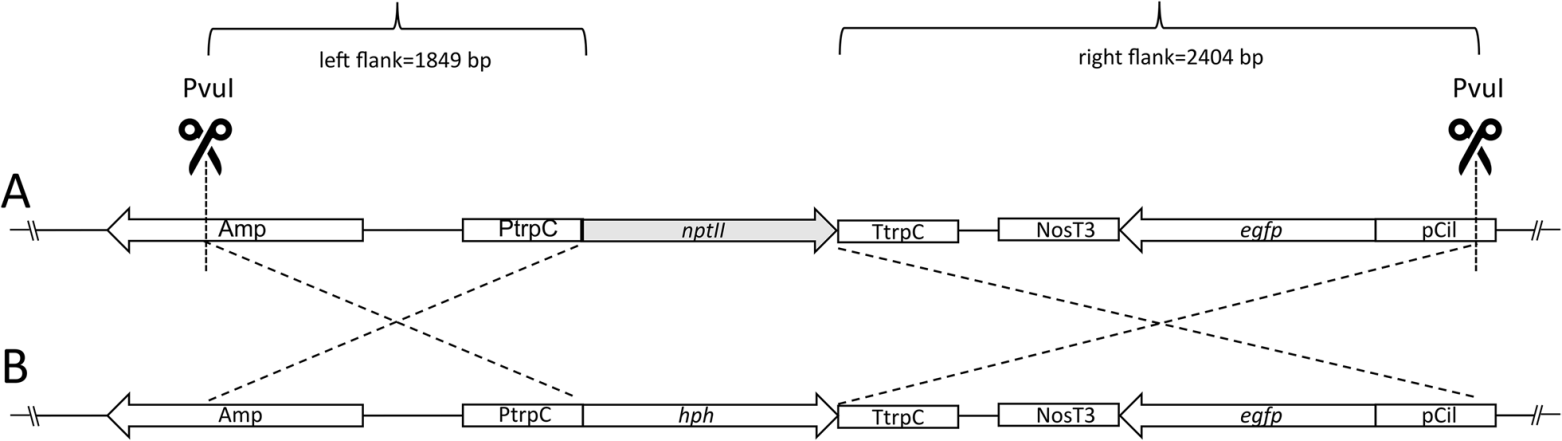
Drawing of the strategy for the replacement of the *hph* gene of mutant #1.12 by the *nptII* gene. **A** The final construct with the *nptII* resistance cassette (pIGPAPA_Gen_GFP) and **B** clipped part of the pIGPAPA_Hyg_GFP. Flanking regions are indicated as crossed boldface black lines. Prior to transformation, the construct was excised PvuI from the plasmid pIGPAPA_Gen_GFP

For overexpression of the enhanced green fluorescence protein (GFP) under the control of the *Neurospora crassa* isocitrate lyase gene promoter, the plasmid pIGPAPA (pIGPAPA_Hyg_GFP) was used [21]. For overexpression of mCherry under the control of the *Aspergillus nidulans* glyceraldehyde-3-phosphate dehydrogenase promoter, the plasmid pAN_Hyg_mCherry (unpublished, Additional file 1: Fig. S1) was used. Transformants of both constructs were selected by the expression of the *hph* gene (hygromycin resistance cassette) under the control of the *Aspergillus nidulans* TrpC promoter. To replace the hygromycin resistance with the geneticin resistance by homologous recombination, pIGPAPA_Hyg_GFP was modified (Fig. 1). The *nptII* gene including the *Aspergillus nidulans* TrpC promoter was excised from plasmid pII99 [22] XhoI/EcoRV and ligated into the XhoI/KspAI opened pIGPAPA_Hyg_GFP to obtain pIGPAPA_Gen_GFP.

Prior to the transformation, the plasmids were linearized and the restriction enzyme activity was inactivated. The construct pIGPAPA_Hyg_GFP was digested with CaiI and pAN_Hyg_mCherry SmaI. For the replacement of the *hph* gene with the *nptII* gene by homologous recombination, pIGPAPA_Gen_GFP was digested PvuI to obtain a 5048 bp fragment including the *nptII* gene flanked by 1849 nts upstream and 2404 nts downstream. Upstream and downstream, flanking regions were homologous to the respective regions of the *hph* gene of the plasmid pIGPAPA_Hyg_GFP.

### Sensitivity to antibiotics

To test the sensitivity of *H. fraxineus* NW-FVA 1856 to hygromycin and geneticin for the subsequent selection of transformants, growth was tested on AM_S_ containing increasing concentrations of the respective antibiotics using either mycelium plugs or protoplasts. For hygromycin (hygromycin B, Duchefa Biochemie), concentrations ranging from 25 to 200 µg/ml and for geneticin (geneticin disulphate (G418) solution, BioScience Grade, Carl Roth GmbH, Karlsruhe, Germany), concentrations in the range of 75–500 µg/ml were tested.

Single mycelium plugs (ø 0.3 cm) of *H. fraxineus* NW-FVA 1856 grown on AM_S_ for 14 days at RT were inoculated on petri dishes (ø 6 cm) containing 5 ml of AM_S_ excluding or including antibiotics with above mentioned concentrations. Plates were incubated at RT in the dark and the growth of mycelium was documented after 21 days.

### Evaluation of enzymes for the generation of protoplasts

All steps were performed under aseptic conditions. To obtain protoplasts, Driselase, Lysing Enzymes or chitinase were prepared in MgSO_4_-buffer at pH 3.5, 5.0 and 5.8 (Table 1) and were tested for their efficiency to digest the fungal cell wall. For this, a culture of *H. fraxineus* NW-FVA 1856 was rejuvenated twice as described in the “Fungal strain and culture conditions” section and incubated for 3 days before the mycelium was harvested by centrifugation (10 min, 2000×*g*). The mycelium was washed by resuspending it in 50 ml ddH_2_O and pelleting by centrifugation (10 min, 2000×*g*). The supernatant was discarded and the mycelium in the pellet was resuspended in 30 ml ddH_2_O, before aliquots of 10 ml were transferred to 15 ml reaction tubes. After centrifugation (10 min, 2000×*g*), the mycelium was resuspended in 5 ml of each enzyme solution and incubated for 16 h at 30 °C in the dark, shaking at 50 rpm. Protoplasts were separated from undigested mycelium by filtration through a 100 µm sieve (Easystrainer for 50 ml tubes, Greiner Bio-One, Frickenhausen, Germany). To collect the protoplasts, the filtrate was mixed with 5 ml 850 mM sucrose in ddH_2_O and overlaid with 200 µl STC (Table 1). During centrifugation (20 min, 2000×*g*), the protoplasts migrated into the top layer, from which 400 µl were collected with a cut 1 ml tip. The protoplasts therein were counted using a Neubauer chamber. To test their ability to regenerate, protoplasts were incubated in AM_RegL_ (Table 1) for 4 days and monitored for the emergence of hyphae with a stereo magnifier (AZ100, Nikon, Minato, Japan) using brightfield.

### Generation of protoplasts for transformation

The testing of the enzyme solution for efficient protoplasting revealed that Driselase worked best and was therefore used to obtain protoplasts for further experiments. Each step was performed at RT under aseptic conditions. In the following paragraphs, the final protocol for protoplasting for transformation is described.

A rejuvenated culture was grown for 3 days as described in the “Fungal strain and culture conditions” section. The mycelium was harvested (10 min, 2000×*g*) and the pellets were washed with 20 ml ddH_2_O by vigorous shaking and centrifuged again (10 min, 2000×*g*). The washing was repeated twice after the volume of the resuspended mycelium was adjusted to 50 ml with ddH_2_O. After the last washing step, the remaining mycelium was resuspended in 5 ml Driselase and incubated 16 h at 30 °C shaking at 50 rpm.

Protoplasts were separated from undigested mycelium by filtering through a 100 µm sieve (Easystrainer for 50 ml tubes, Greiner Bio-One). Subsequently, the protoplast suspension was adjusted to 10 ml with MgSO_4_-buffer and mixed with 10 ml 850 mM sucrose in ddH_2_O. The suspension was overlayed with 400 µl STC (Table 1) and centrifuged for 20 min at 2000×*g*. From the top, 600 µl of the floating protoplasts were collected with a cut 1 ml tip.

### Transformation

An aliquot of 300 µl protoplast suspension (containing a minimum of 1 × 10^5^ protoplasts) was carefully mixed in a 50 ml reaction tube with linearized plasmid (1–10 µg in 30 µl) and incubated for 10 min at RT. The suspension was diluted with 1 ml PEG/STC (Table 1), incubated (10 min at RT) and gently mixed with 5 ml AM_RegL_. The cells were regenerated for 3 days in the dark at RT.

Regenerated protoplasts were mixed with 45 ml AM_RegS_ (< 50 °C, Table 1) and aliquots of 10 ml were transferred to petri dishes (ø 9 cm). After 4 days of incubation at RT in the dark, the cultures were overlayed with 10 ml of Water agar (Table 1) including 200 µg/ml hygromycin (pIGPAPA_Hyg_GFP, pAN_Hyg_mCherry) or 750 µg/ml geneticin (pIGPAPA_Gen_GFP), respectively, to obtain a final concentration in AM_RegS_ + overlay of 100 µg/ml hygromycin or 375 µg/ml geneticin, in the total of the 20 ml medium in each plate. After 2 to 10 days of incubation in the dark at RT, transformants emerging through the antibiotic layer were transferred to AM_HygS_ or AM_GenS_ (Table 1).

### Screening for the expression of the reporter genes in vitro

First, putative transformants, which were transferred from the selection plates to AM_s_, were visually screened for the expression of the respective reporter gene with a Leica Microscope MZFLIII (Leica Microsystems, Heerbrugg, Switzerland) using the DsRed filter set for mCherry detection containing an excitation filter at 546/12 nm and a long pass filter at 560 nm. For GFP detection, the GFP3 filter set with an excitation filter at 470/40 nm and a long pass filter at 510 nm was used. Additionally, fluorescence was detected using the Axio Imager Z1 (Zeiss, Oberkochen, Germany). DsRed was excited in the range of 538–562 nm and detected in the 570–640 nm range. GFP was excited with 450–490 nm and detected at 500–550 nm. Images were taken with an AxioCam MRm CCD (Zeiss) camera. Image processing was performed with Zeiss AxioVision software (version 4.8.2.0).

### Screening for stable transformants

The stable integration of DNA into the fungal genome was verified by Southern Blot or PCR. For that, gDNA was extracted with modifications [23]. Approximately 100 mg of semi-dried mycelium was crushed in liquid nitrogen. The resulting powder was resuspended in 900 μl CTAB-buffer (Table 1) followed by incubation at 65 °C for 1 h. The suspension was centrifuged to remove coarse material (14,000×*g*, 2 min) and the cleared supernatant was extracted once with 900 μL chloroform. The DNA in the upper phase was precipitated with 750 μL isopropyl alcohol (30 min, − 20 °C), pelleted (14,000×*g*, 30 min, 4 °C), washed with 70% (v/v) EtOH and dried. The pellet was resuspended in 450 μl TE-buffer/RNase (Table 1) at 55 °C shaking at 300 rpm for 16 h.

Southern Blot was performed as described by Salomon et al. [24]. Approximately 3 µg of the gDNA was digested HindIII (integrated construct: pIGPAPA_Hyg_GFP), EcoRV (integrated construct: pAN_Hyg_mCherry) or HindIII for the knock-out construct (pIGPAPA_Gen_GFP), and separated on a 0.8% (w/v) agarose gel by electrophoresis at 100 V for 3 h. The DNA was transferred by capillary blotting onto a Hybond NX membrane (GE Healthcare, Munich, Germany) and hybridized with a DIG (digoxigenin)-labeled DNA probe. All probes were amplified by PCR using DIG-UTP (Roche, Penzberg, Germany) according to the manufacturer´s protocol.

The probe to detect the stable integration of pAN_Hyg_mCherry covered a 452 bp fragment of the hygromycin resistance cassette and was amplified from pIGPAPA_Hyg_GFP using the primer pair 5′-agttcagcgagagcctgaccta-3′/5′-gatgttggcgacctcgtatt-3′. The detection of the stable integration of pIGPAPA_Hyg_GFP was performed with a 532 bp GFP probe, amplified from pIGPAPA_Hyg_GFP with primer pair 5′-gaccaccttcacctacggc-3′/5′-acttgtacagctcgtcca-3′. For detection and visualization of bands by means of CSPD (Roche) and a LAS-3000 imager (Fujifilm Photo Film Co., LTD, Tokyo, Japan), the manufacturers’ protocols were followed.

The replacement of the *hph* gene with the *nptII* gene by homologous recombination with pIGPAPA_Gen_GFP was verified by PCR using the primer pair 5′-agttcagcgagagcctgaccta-3′/5′-gatgttggcgacctcgtatt-3′ to amplify a 452 bp of the *hph* gene. A PCR fragment covering 421 bp of the *nptII* gene was amplified using primer pair 5′-gaagggactggctgctattg-3′/5′-aatatcacgggtagccaacg-3′.

### Detection of a GFP expressing *H. fraxineus* strain on ash seedlings

To establish in vitro cultures of ash seedlings, a modified method of Junker et al. [25] and Raquininsta et al. [26] was employed using ash tree seeds from 2003 (Niedersächsische Landesforsten, Oerrel, from Ostholstein, Friederikenhof, Germany, D-01 001 1 0059 03). After developing leaves and roots, the seedlings were transplanted into baby food jars containing H_10_ medium [26]. Each seedling was inoculated with a small amount of mycelium of the GFP-overexpressing mutant #1.10 or the wild type (wt) strain *H. fraxineus* NW-FVA 1856 at the base of the seedling’s stem and cultivated for 6 weeks. Roots of seedlings were embedded in 10% (w/v) agarose and 40 µm longitudinal sections of root tissue were prepared using a Hyrax V50 (Zeiss). Sections were screened for the presence of mycelium by using the Axio Imager Z1 as described above.

## Results

### Resistance to antibiotics

**Fig. 2:**
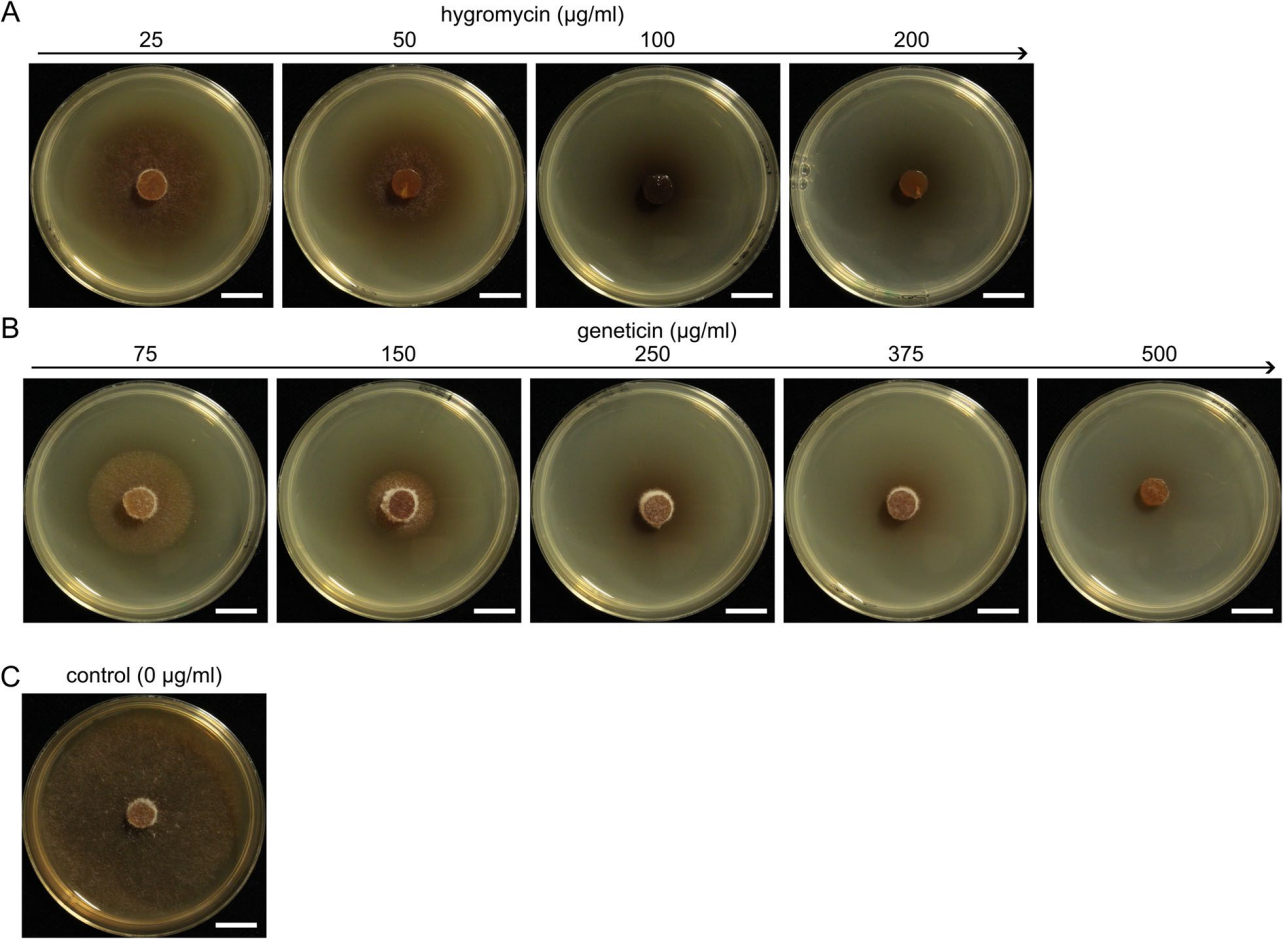
Testing of agar plugs of *H. fraxineus* NW-FVA 1856 for sensitivity to hygromycin **A** and geneticin **B**. Mycelium was inoculated on AM_S_ containing increasing concentrations of antibiotics. **C** *H. fraxineus* NW-FVA 1856 on AM_S_. The growth of mycelium was monitored after 21 days of incubation at RT in the dark. Scale bar = 1 cm

Agar plugs of mycelium of *H. fraxineus* NW-FVA 1856 were inoculated on plates containing AM_S_ with different concentrations of hygromycin (25, 50, 100 and 200 µg/ml) and geneticin (75, 150, 250, 375 and 500 µg/ml), respectively (Fig. 2). On antibiotic-free AM_s_, the complete surface was covered by mycelium after 21 days of incubation. The mycelial growth was directly negatively correlated to increasing concentrations of the respective antibiotic. On plates containing 50–200 µg/ml hygromycin and 375 to 500 µg/ml geneticin, respectively, the growth was completely inhibited. When germinated protoplasts instead of agar plugs were used, the growth was completely inhibited from 50 µg/ml of hygromycin or from 250 µg/ml of geneticin (Additional file 1: Fig. S2). Subsequently, for the selection of transformants, the 10 ml overlay contained 200 µg/ml hygromycin (to be diluted to 100 µg/ml with 10 ml AM_RegS_) or 750 µg/ml geneticin (to be diluted to 375 µg/ml with 10 ml AM_RegS_).

### Evaluation of enzymes for the generation of protoplasts

In preliminary experiments, the 3 enzyme solutions containing Driselase, Lysing Enzymes or chitinase were tested in MgSO_4_-buffer at different pH values (3.5. 5.0 and 5.8) for their suitability for generating protoplasts. Irrespective of the pH value of the MgSO_4_-buffer, chitinase did not digest the mycelium and no protoplasts were obtained. Protoplasts were obtained with both, Driselase and Lysing Enzymes, irrespective of the pH of the MgSO_4_-buffer.

**Fig. 3:**
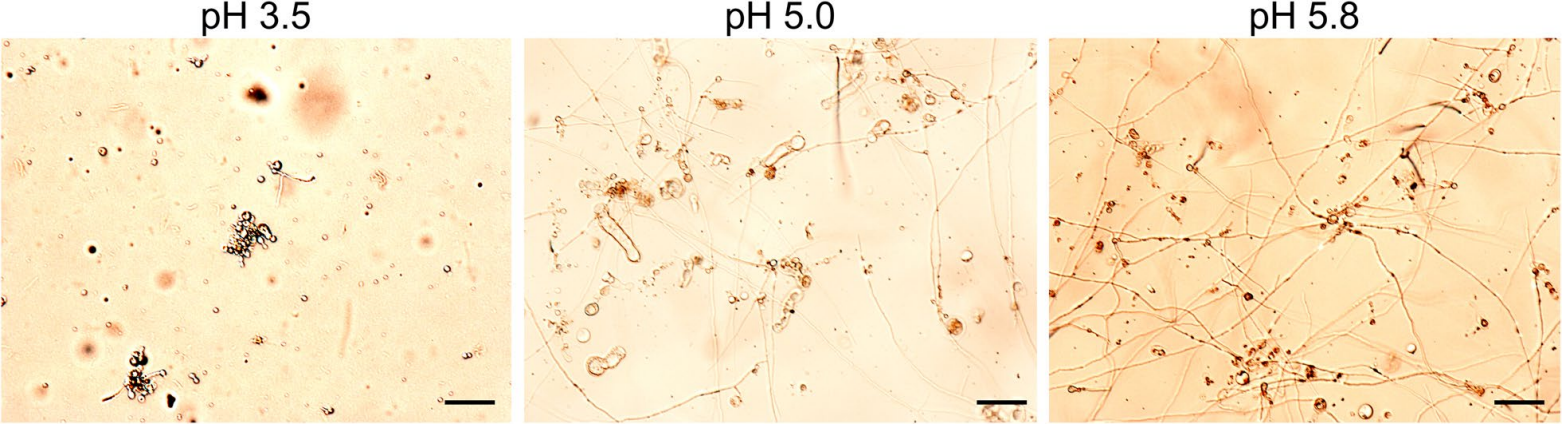
Regeneration of protoplasts to mycelium using Driselase, prepared in MgSO_4_-buffer at pH 3.5, 5.0 and 5.8 and AM_RegL_ at pH 3.5, 5.0 and 5.8, monitored after 4 days. Scale bar = 100 µm

To test the competence for regeneration, protoplasts were incubated in AM_RegL_ with the respective pH values of 3.5, 5.0 and 5.8 and the regeneration was monitored microscopically. Poor regeneration was at pH 3.5, the increase to pH 5.0 led to more regenerated hyphae. The highest proportion of regeneration was obtained with the preparation of AM_RegL_ at pH 5.8 (Fig. 3). Therefore, a comparison of Driselase and Lysing Enzymes for their efficiency to generate protoplasts was performed in 3 independent experiments prepared only in MgSO_4_-buffer at pH 5.8 and AM_RegL_ at pH 5.8.

Using Driselase, a total of 7.5 × 10^5^ to 8 × 10^5^ protoplasts were obtained, while the use of Lysing Enzymes resulted in a range of 2.4 × 10^5^ to 4.6 × 10^5^ protoplasts (Table 2).

**Table 2:** Number of protoplasts prepared with driselase and lysing enzymes in MgSO_4_-buffer (pH 5.8)

	Experiment 1	Experiment 2	Experiment 3
Driselase	7.5 × 10^5^	8.4 × 10^5^	8.0 × 10^5^
Lysing enzymes	4.2 × 10^5^	4.6 × 10^5^	2.4 × 10^5^

### Transformation

**Fig. 4:**
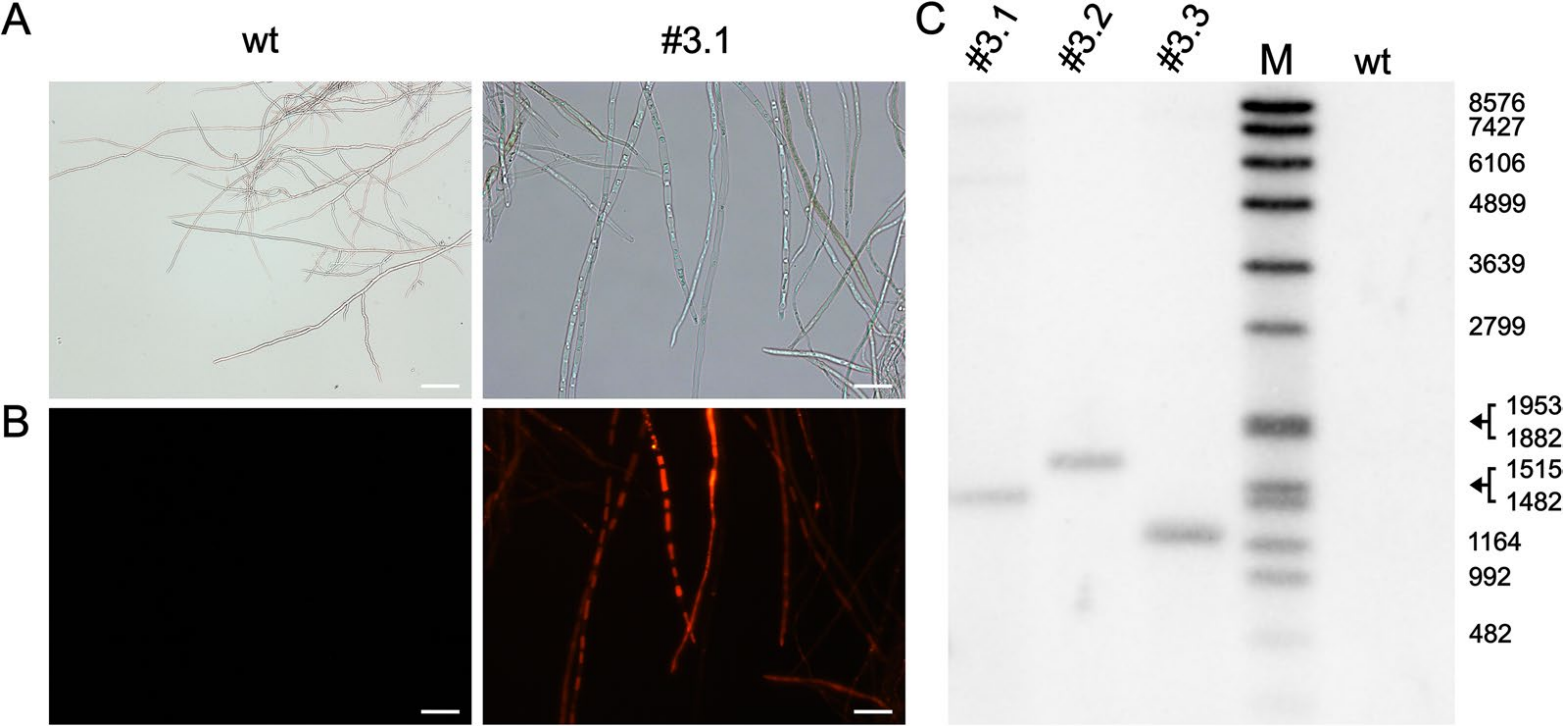
Transformants expressing mCherry after transformation with pAN_Hyg_mCherry. **A** Brightfield of the hyphae and **B** mCherry flurorescence of isolate #3.1. Scale bar = 20 µm. **C** Southern Blot of transformants #3.1, #3.2 and #3.3. wt = *H. fraxineus* NW-NVA 1856. M = DNA Molecular Weight Marker VII (Roche). Sizes of the marker bands are displayed in bp on the right

In the first experiment, protoplasts of *H. fraxineus* NW-FVA 1856 were transformed with pAN_Hyg_mCherry and 3 mutants were obtained. All mutants showed similar mCherry expression (Fig. 4A and B) and stable integration of the construct was verified by Southern Blot, detecting the hygromycin resistance cassette (Fig. 4C). Due to the linearization with SmaI of the construct prior to transformation and the digestion of gDNA with EcoRV, bands with sizes of more than 1291 bp were expected. Single bands in the range of larger than 1164 bp and smaller than 1953 bp were detected, suggesting single integration of the construct in each mutant. In 11 more independent experiments, 2 to 68 transformants were obtained (Additional file 1: Table S1).

**Fig. 5:**
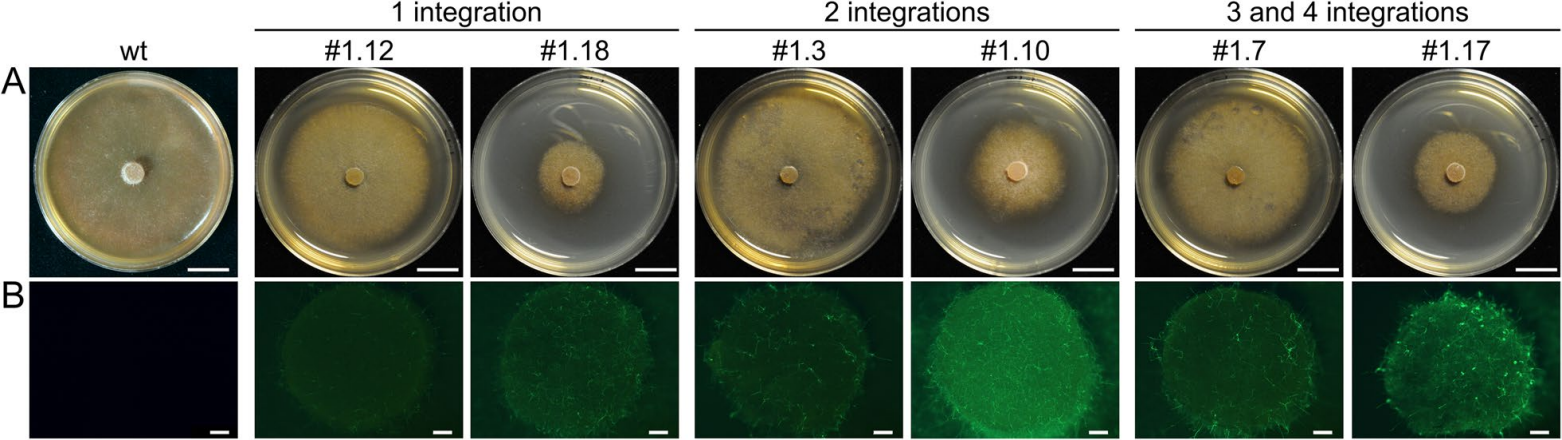
Number of integrations in relation to the level of GFP expression after 7 days of growth on AM_S_. Numbers of integrations and the respective denomination of the mutants are displayed on top. **A** Growth recorded with brightfield. Scale bar = 1 cm. **B** Expression of GFP recorded at 470/40 nm. Scale bar = 1 mm. wt = *H. fraxineus* NW-FVA 1856

After transformation of *H. fraxineus* NW-FVA 1856 with pPIGPAPA_Hyg_GFP, 29 single colonies were transferred to AM_HygS_ and all of them grew, suggesting successful transformation and expression of the resistance gene. From these, 19 putative mutants showed varying GFP expression. Ten mutants with different expression levels were selected to determine the number of integrations by Southern Blot, detecting the GFP gene. Due to the linearization of the plasmid with CaiI prior to transformation, the digestion of the gDNA with HindIII and the random locus integration of the construct into the genome, bands with a minimum size of 2295 bp were expected. All tested transformants revealed that the construct had been stably integrated 1–4 times (Additional file 1: Fig. S3). The number of integrations did not correlate with the level of GFP fluorescence (Fig. 5).

### Live cell imaging of roots of inoculated ash seedlings

**Fig. 6:**
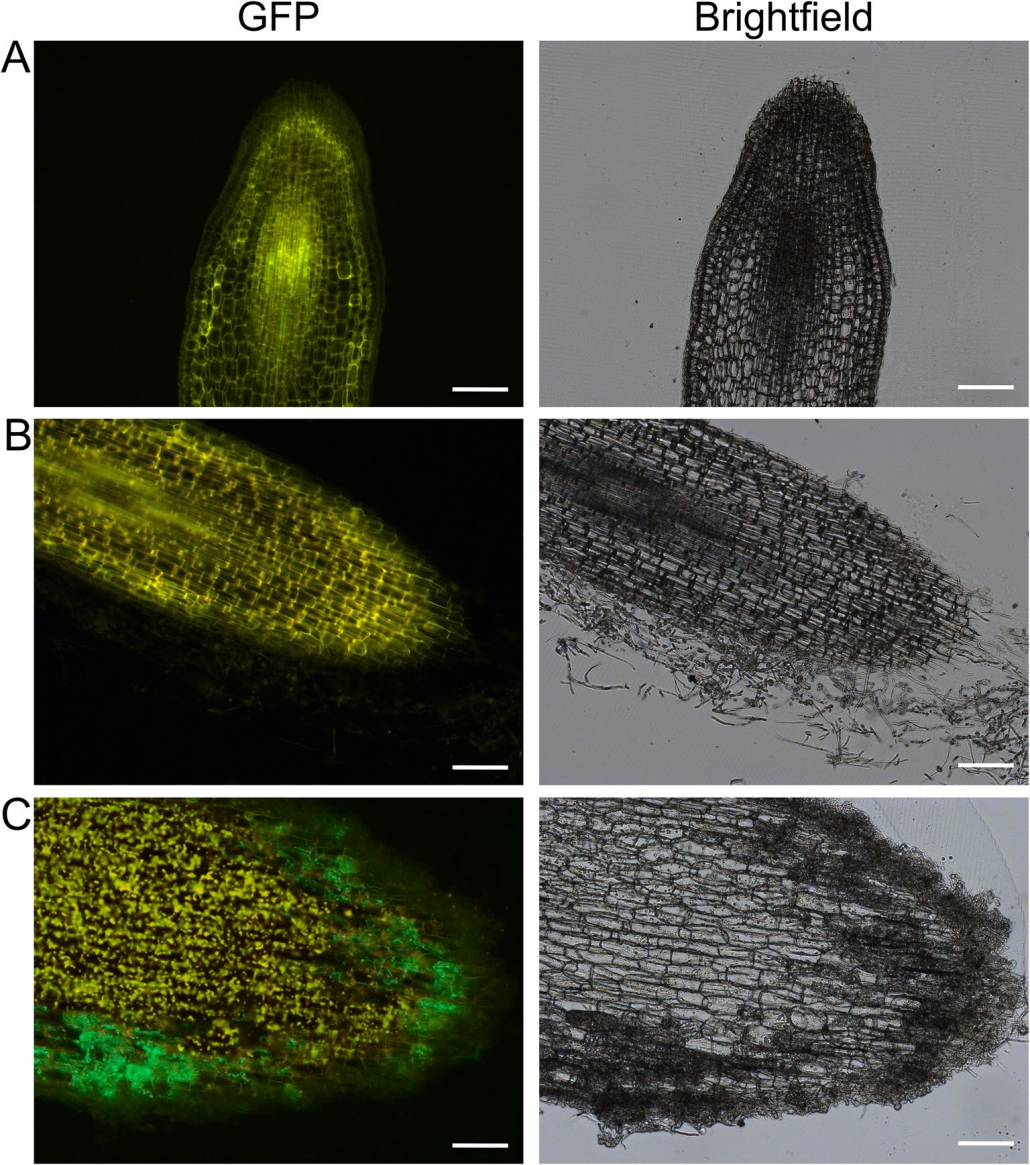
Root tip of ash seedlings 6 weeks post inoculation in longitudinal sections of 40 µm thickness in fluorescence- and brightfield microscopy. **A** Inoculation with the water control. Scale bar = 200 µm. **B** Inoculation with wt *H. fraxineus* NW-FVA 1856. Scale bar = 200 µm. **C** Inoculation with isolate #1.10. Scale bar = 50 µm

Ash seedlings, which were grown in vitro, were infected with the wt *H. fraxineus* NW-FVA 1856 and the GFP expressing strain #1.10. After 6 weeks of incubation, seedlings were explanted and root tissue was screened for the presence of mycelium. Longitudinal sections of root tips of 40 µm were further analyzed microscopically (Fig. 6). The center of the sections showed yellow autofluorescence while mycelium-expressing GFP was detected in green (Fig. 6C).

### Gene replacement by homologous recombination

**Fig. 7:**
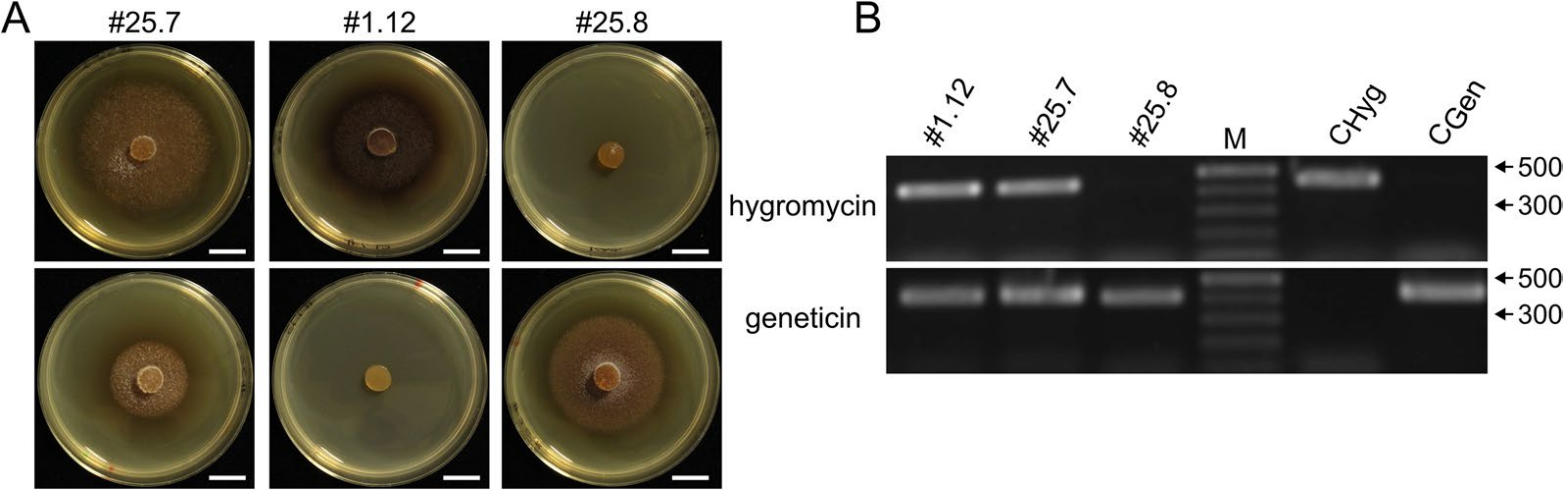
Verification of the replacement of the hygromycin resistance by a geneticin resistance in mutant #1.12 via homologous recombination. **A** Plate assay. Mutant #25.7 grew on both selectable mediums, mutant #1.12 grew on AM_HygS_ but not on AM_GenS_ while mutant #25.8 grew on AM_GenS_ but not on AM_HygS_. Scale bar = 1 cm. **B** PCR to detect the *hph* gene (expected band size is 452 bp) and the *nptII* gene (expected band size is 421 bp). In mutant #1.12 only a fragment of the *hph* gene and in mutant #25.8 only a fragment of the *nptII* gene was detected. In mutant #25.7, both genes were detected. M = GeneRuler^TM^1kb Plus DNA Ladder (ThermoFisher, Waltham, USA). Sizes of the marker bands are displayed in bp at the right. C_Hyg_ = positive control pAN_Hyg_mCherry, C_Gen_ = positive control pIGPAPA_Gen_GFP

As a proof of principle for targeted gene knock-out, the *hph* gene was replaced by the *nptII* gene via homologous recombination. For that, the GFP expressing mutant #1.12 (single integration of pIGPAPA_Hyg_GFP) was transformed with pIGPAPA_Gen_GFP. In total, 39 transformants were obtained and were screened for both, hygromycin and geneticin resistance on AM_HygS_ and AM_GenS_. One transformant (#25.8) was obtained that only grew on AM_GenS_, suggesting integration into the correct locus (Fig. 7A). The replacement was further confirmed by PCR and agarose gel electrophoresis (Fig. 7B). While the putative knock-out mutant #25.8 showed the expected band size of 421 bp confirming the integration of the *nptII* gene, no amplicon for the *hph* gene was detected. A fragment of 452 bp was amplified from mutant #1.12, which was used as primary isolate for transformation, confirming the presence of the *hph* gene. Mutant #25.7 showed an amplicon for each primer pair and it grew on both, AM_GenS_ and AM_HygS_, suggesting an ectopic integration of the construct.

## Discussion

Based on the studies of Li et al. [16] and Monma et al. [27] in combination with a protocol used for *Fusarium graminearum* (*F. graminearum*) transformation [28], we developed a protocol for the genetic manipulation of *H. fraxineus*.

The main challenge for protoplasting was the choice of the starting material. Many protocols, developed for protoplast preparation, utilize mycelium of germinated spores as it was described for *F. graminearum* by Maier et al. [29]. This material provides a synchronous culture with thin cell walls and with a similar physiology and biochemistry. When older mycelium is used, it can result in asynchronous cells which alter according to their differentiation [17]. *Hymenoscyphus fraxineus* produces a high number of spermatia. However, since their germination rate is extremely low [13], mycelium that emerges from those spores does not provide a sufficient amount for the generation of protoplasts for further transformation. To obtain yet a maximum number of cells with a comparable physiology, mycelial cultures were rejuvenated twice after storage. The rejuvenation of the culture by shredding and subsequent incubation for 3 days provided suitable starting material for obtaining a high number of protoplasts.

The choice of enzymes for protoplasting of filamentous fungi depends on their species and state of growth which has to be tested individually and is therefore a key factor for protoplasting (reviewed in Li et al. [16]). While for some fungi Driselase does not work [30], it results in the highest number of protoplasts for other fungi [31]. For the preparation of protoplasts of *H. fraxineus*, Driselase was also superior in 3 independent experiments in comparison to Lysing Enzymes. Nevertheless, in summary, both, Driselase and Lysing Enzymes are suitable for protoplasting *H. fraxineus.* As a low-cost option, we also tested chitinase, but observed no cell wall digestion.

As osmotic stabilizers, inorganic (Mg_2_SO_4_, KCl, NaCl) and organic (mannitol, sorbitol, sucrose) chemicals at various concentrations are commonly tested for their impact on protoplast number, size and appearance. Here, we tested Mg_2_SO_4_ since it was discussed by Fariña et al. [32] to be most effective for protoplast isolation for several filamentous fungi. Following Wu et al. [33], we used 1 M Mg_2_SO_4_ for protoplasting. Although Mg_2_SO_4_ at pH 3.5, 5.0 and 5.8 resulted in similar numbers of protoplasts, solely protoplasts produced in Mg_2_SO_4_ at pH 5.8 regenerated to mycelium efficiently.

While protoplasts of *F. graminearum* can be pelleted by centrifugation, protoplasts of *H. fraxineus* did not pellet. However, for protoplast collection, we found that they concentrated in an overlay with reduced sucrose concentration as free-floating cells.

For the selection of transformed cells, the right antibiotics need to be selected and their concentrations needed to be optimized. A hygromycin resistance, which is induced by the expression of the *hph* gene, is the most commonly used selection marker in the transformation of filamentous fungi [34]. We found that not only hygromycin resistance mediated selection works for the selection of transformed *H. fraxineus*, but also a geneticin resistance, induced by the expression of the *nptII* gene. However, it is important to note that the concentrations of both antibiotics varied when using mycelium or protoplasts. Also, it is possible that other strains of *H. fraxineus* are tolerant to different concentrations of antibiotics and should be tested prior to transformation individually.

For the overexpression of reporter genes, we used the same promoters as we did for the overexpression in *F. graminearum* [28]. We produced mutants with up to 4 integrations and found no correlation with the level of the reporter gene expression. The differences in gene expression may be attributed to the locus of integration. This is in accordance with Wu et al. [35] who showed for yeast that not the number of integrations, but rather the locus on the chromosomal DNA is crucial for the level of reporter gene expression. The level of expression was suitable for monitoring mycelium which had been inoculated into ash seedlings in vitro. Such overexpression mutants may help to further elucidate the fungal biology of *H. fraxineus* and its interaction with its host.

Further, we showed as a proof of principle that targeted gene manipulation was achieved by homologous recombination. The *hph* gene was successfully replaced with the *nptII* gene using flanking regions on each side of the targeted locus, although the frequency of the correct integration was low. Further modification of the construct may be required to achieve a higher efficiency.

This study is the first report of successful protoplast generation and stable transformation of *H. fraxineus*. The transformants can be used for studying the fungus’ colonization in plants. Furthermore, the protocol described here enables targeted gene deletion for functional analysis to elucidate the pathogenic mechanisms of the fungus.

## Conclusions

The invasive fungal pathogen *H. fraxineus* threatens the European ashes for which a pest control management has yet to be developed. For that, a better understanding of the fungal biology and the interaction with its host is required. The expression of reporter genes for in situ monitoring of fungal colonization of the host as well as targeted gene knock-out are state of the art techniques to accomplish these goals. In our study we not only developed a protocol for stable transformation of *H. fraxineus* to obtain fluorescence reporter strains but also, we showed that the mycelium of the fluorescence reporter mutant is detectable in infected plant tissue. In addition, we showed as a proof of principle that targeted gene knock-out is possible by homologous recombination. For the development of the transformation 2 key problems were solved. First, the starting material was obtained by rejuvenation of the culture by shredding, and second, collection of protoplasts was obtained as floating cells. Our study will help to alleviate future investigations of the biology of *H. fraxineus* and may contribute to the development of a pest management strategy to prevent the loss of the European ashes.

## Supplementary Information

**Additional file 1: Figure S1.** Map and sequence of the construct pAN_Hyg_mCherry. The restriction site SmaI was used for linearization prior to transformation. **Figure S2.** Testing of germinating protoplasts of *H. fraxineus* NW-FVA 1856 for their sensitivity to hygromycin (**A**) and geneticin (**B**). Mycelium was inoculated on AM_RegS_ containing increasing concentrations of antibiotics. The growth of mycelium was monitored after 21 days of incubation at RT in the dark. Scale bar = 1 cm. **Table S1.** Obtained mutants after eleven independent transformations. **Figure S3.** Detection of the number of integrations of pPIGPAPA_Hyg_GFP into the genome of *H. fraxineus* NW-FVA 1856. Three transformants (#1.12, #1.18 and #1.19) had a single integration, 3 transformants had a double integration (#1.3, #1.10, #1.16) and in 4 transformants, the construct was integrated 3 or 4 times (#1.1, #1.6, #1.7, #1.17). wt= wild type, M=DNA Molecular Weight Marker VII (Roche Penzberg, Germany). The sizes of the marker bands are shown in bp on the left.

## Data Availability

All data generated or analyzed during this study are included in this published article and its additional files.

## Abbreviations

AM_GenS_: Solid ash medium with geneticin
AM_HygS_: Solid ash medium with hygromycin
AM_L_: Liquid ash medium
AM_RegL_: Liquid ash medium for regeneration
AM_RegS_: Solid ash medium for regeneration
AM_S_: Solid ash medium
ddH_2_O: Double distilled H_2_O
GFP: Enhanced green fluorescence protein
RT: Room temperature
wt: Wild type
