# Supplementary material for: Stable overexpression and targeted gene deletion of the causative agent of ash dieback Hymenoscyphus fraxineus
Source: Fungal Biol Biotechnol. 2023 Jan 13;10:1. doi: 10.1186/s40694-023-00149-y (PMC9840287; doi:10.1186/s40694-023-00149-y)
Supplement: Supplementary file 1 — Additional file 1: Figure S1. Map and sequence of the construct pANHygmCherry. The restriction site SmaI was used for linearization prior to transformation. Figure S2. Testing of germinating protoplasts of H. fraxineus NW-FVA 1856 for their sensitivity to hygromycin (A) and geneticin (B). Mycelium was inoculated on AMRegS containing increasing concentrations of antibiotics. The growth of mycelium was monitored after 21 days of incubation at RT in the dark. Scale bar = 1 cm. Table S1. Obtained mutants after eleven independent transformations. Figure S3. Detection of the number of integrations of pPIGPAPAHygGFP into the genome of H. fraxineus NW-FVA 1856. Three transformants (#1.12, #1.18 and #1.19) had a single integration, 3 transformants had a double integration (#1.3, #1.10, #1.16) and in 4 transformants, the construct was integrated 3 or 4 times (#1.1, #1.6, #1.7, #1.17). wt= wild type, M=DNA Molecular Weight Marker VII (Roche Penzberg, Germany). The sizes of the marker bands are shown in bp on the left. [file 40694_2023_149_MOESM1_ESM.docx]

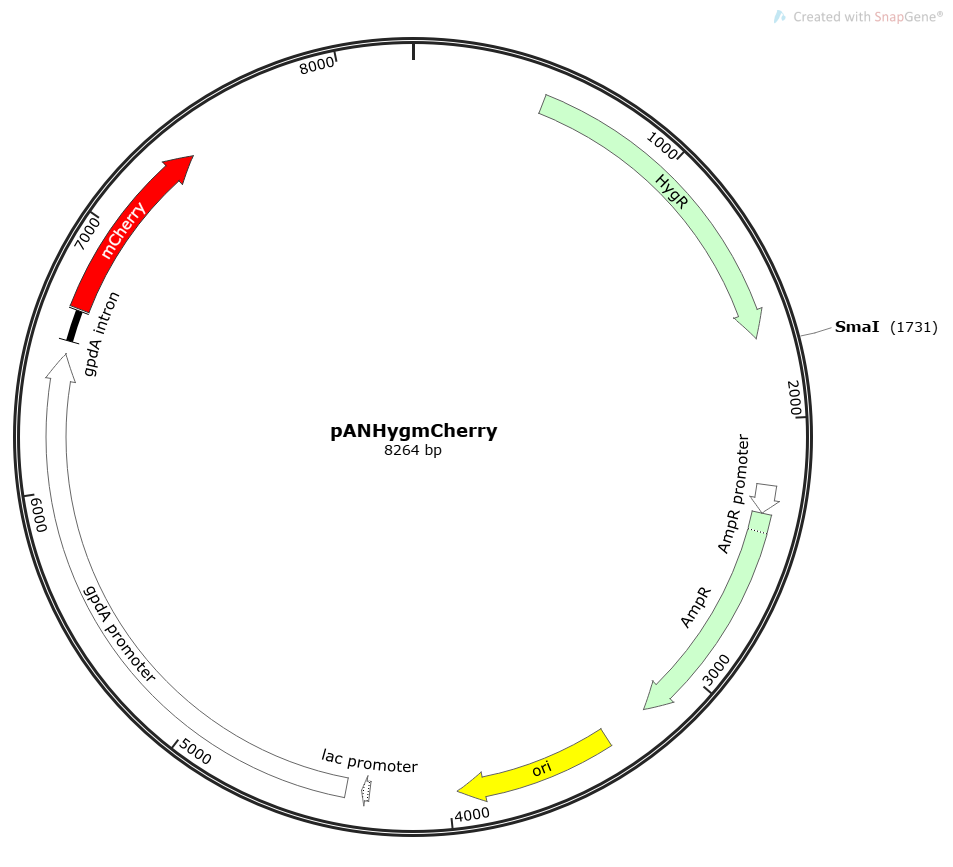
 CTAGTGTACCACTTGACCAAAAGGTACCCAGGCTGACCTTCACGCCCTCCGGTGCTCTCCGCGGCTTCGAATCGTGGCTACCCTTGGCCGAGGTTGCGATTTCTCTGCCGTATCTGACAATGATCCAACCGGCCGGATGTGTGGGAGGGCGCAAAGGGTGTAATTGGGTCTAGGCGTGAGCTGCATGTCCGATGGCAGAAAAAGAACTACTAAAACTCTGTGACGTCCTCAGGGCTGGTAGCTATTTAGCTTGCCCCTCCCTCACCGGACCTAGCTCAAACCCAACAAAACACAGCACCGACCACAAAAATCTGCCTCTTACCACCTGCTCATCACCTTTTCTCACATATAAAGCAGTGAGCTCAGCCCGATTTCCATTCCTCAATTCAAGTCTATTAACTCTCTCAAAGCATCACTCTCAGTGGGGATCCAAGCTCGATATCTAACCCTTTGATATAGTGCAACAGGTGCTCAGCCTACAATCGCCATGAGCGCCCCTACAGATACCACAGACGGCAAGCCCGAGCGACCTTCAATCGCTTCCCAAGAAAGCTCTCACCCTGCACTCCATCCCGACATCACCAACGTACCCGTCACCCCCGGCGTCCACCTCTCGGGCAAGACGGCCTACTTTGACGAGTCCTCCGACAAGAAAGAGGGCAGCAGCCTGCCCTTTGATCCACCTGAACTCACCGCGACGTCTGTCGAGAAGTTTCTGATCGAAAAGTTCGACAGCGTCTCCGACCTGATGCAGCTCTCGGAGGGCGAAGAATCTCGTGCTTTCAGCTTCGATGTAGGAGGGCGTGGATATGTCCTGCGGGTAAATAGCTGCGCCGATGGTTTCTACAAAGATCGTTATGTTTATCGGCACTTTGCATCGGCCGCGCTCCCGATTCCGGAAGTGCTTGACATTGGGGAATTCAGCGAGAGCCTGACCTATTGCATCTCCCGCCGTGCACAGGGTGTCACGTTGCAAGACCTGCCTGAAACCGAACTGCCCGCTGTTCTGCAGCCGGTCGCGGAGGCCATGGATGCGATAGCTGCGGCCGATCTTAGCCAGACGAGCGGGTTCGGCCCATTCGGACCGCAAGGAATCGGTCAATACACTACATGGCGTGATTTCATATGCGCGATTGCTGATCCCCATGTGTATCACTGGCAAACTGTGATGGACGACACCGTCAGTGCGTCCGTCGCGCAGGCTCTCGATGAGCTGATGCTTTGGGCCGAGGACTGCCCCGAAGTCCGGCACCTCGTGCACGCGGATTTCGGCTCCAACAATGTCCTGACGGACAATGGCCGCATAACAGCGGTCATTGACTGGAGCGAGGCGATGTTCGGGGATTCCCAATACGAGGTCGCCAACATCTTCTTCTGGAGGCCGTGGTTGGCTTGTATGGAGCAGCAGACGCGCTACTTCGAGCGGAGGCATCCGGAGCTTGCAGGATCGCCGCGGCTCCGGGCGTATATGCTCCGCATTGGTCTTGACCAACTCTATCAGAGCTTGGTTGACGGCAATTTCGATGATGCAGCTTGGGCGCAGGGTCGATGCGACGCAATCGTCCGATCCGGAGCCGGGACTGTCGGGCGTACACAAATCGCCCGCAGAAGCGCGGCCGTCTGGACCGATGGCTGTGTAGAAGTACTCGCCGATAGTGGAAACCGACGCCCCAGCACTCGTCCGAGGGCAAAGGAATAGAGTAGATGCCGACCGAACAAGAGCTGTCCCCCGGGGGAATCACTAGAGCTTGGCACTGGCCGTCGTTTTACAACGTCGTGACTGGGAAAACCCTGGCGTTACCCAACTTAATCGCCTTGCAGCACATCCCCCTTTCGCCAGCTGGCGTAATAGCGAAGAGGCCCGCACCGATCGCCCTTCCCAACAGTTGCGCAGCCTGAATGGCGAATGGCGCCTGATGCGGTATTTTCTCCTTACGCATCTGTGCGGTATTTCACACCGCATATGGTGCACTCTCAGTACAATCTGCTCTGATGCCGCATAGTTAAGCCAGCCCCGACACCCGCCAACACCCGCTGACGCGCCCTGACGGGCTTGTCTGCTCCCGGCATCCGCTTACAGACAAGCTGTGACCGTCTCCGGGAGCTGCATGTGTCAGAGGTTTTCACCGTCATCACCGAAACGCGCGAGACGAAAGGGCCTCGTGATACGCCTATTTTTATAGGTTAATGTCATGATAATAATGGTTTCTTAGACGTCAGGTGGCACTTTTCGGGGAAATGTGCGCGGAACCCCTATTTGTTTATTTTTCTAAATACATTCAAATATGTATCCGCTCATGAGACAATAACCCTGATAAATGCTTCAATAATATTGAAAAAGGAAGAGTATGAGTATTCAACATTTCCGTGTCGCCCTTATTCCCTTTTTTGCGGCATTTTGCCTTCCTGTTTTTGCTCACCCAGAAACGCTGGTGAAAGTAAAAGATGCTGAAGATCAGTTGGGTGCACGAGTGGGTTACATCGAACTGGATCTCAACAGCGGTAAGATCCTTGAGAGTTTTCGCCCCGAAGAACGTTTTCCAATGATGAGCACTTTTAAAGTTCTGCTATGTGGCGCGGTATTATCCCGTATTGACGCCGGGCAAGAGCAACTCGGTCGCCGCATACACTATTCTCAGAATGACTTGGTTGAGTACTCACCAGTCACAGAAAAGCATCTTACGGATGGCATGACAGTAAGAGAATTATGCAGTGCTGCCATAACCATGAGTGATAACACTGCGGCCAACTTACTTCTGACAACGATCGGAGGACCGAAGGAGCTAACCGCTTTTTTGCACAACATGGGGGATCATGTAACTCGCCTTGATCGTTGGGAACCGGAGCTGAATGAAGCCATACCAAACGACGAGCGTGACACCACGATGCCTGTAGCAATGGCAACAACGTTGCGCAAACTATTAACTGGCGAACTACTTACTCTAGCTTCCCGGCAACAATTAATAGACTGGATGGAGGCGGATAAAGTTGCAGGACCACTTCTGCGCTCGGCCCTTCCGGCTGGCTGGTTTATTGCTGATAAATCTGGAGCCGGTGAGCGTGGGTCTCGCGGTATCATTGCAGCACTGGGGCCAGATGGTAAGCCCTCCCGTATCGTAGTTATCTACACGACGGGGAGTCAGGCAACTATGGATGAACGAAATAGACAGATCGCTGAGATAGGTGCCTCACTGATTAAGCATTGGTAACTGTCAGACCAAGTTTACTCATATATACTTTAGATTGATTTAAAACTTCATTTTTAATTTAAAAGGATCTAGGTGAAGATCCTTTTTGATAATCTCATGACCAAAATCCCTTAACGTGAGTTTTCGTTCCACTGAGCGTCAGACCCCGTAGAAAAGATCAAAGGATCTTCTTGAGATCCTTTTTTTCTGCGCGTAATCTGCTGCTTGCAAACAAAAAAACCACCGCTACCAGCGGTGGTTTGTTTGCCGGATCAAGAGCTACCAACTCTTTTTCCGAAGGTAACTGGCTTCAGCAGAGCGCAGATACCAAATACTGTCCTTCTAGTGTAGCCGTAGTTAGGCCACCACTTCAAGAACTCTGTAGCACCGCCTACATACCTCGCTCTGCTAATCCTGTTACCAGTGGCTGCTGCCAGTGGCGATAAGTCGTGTCTTACCGGGTTGGACTCAAGACGATAGTTACCGGATAAGGCGCAGCGGTCGGGCTGAACGGGGGGTTCGTGCACACAGCCCAGCTTGGAGCGAACGACCTACACCGAACTGAGATACCTACAGCGTGAGCTATGAGAAAGCGCCACGCTTCCCGAAGGGAGAAAGGCGGACAGGTATCCGGTAAGCGGCAGGGTCGGAACAGGAGAGCGCACGAGGGAGCTTCCAGGGGGAAACGCCTGGTATCTTTATAGTCCTGTCGGGTTTCGCCACCTCTGACTTGAGCGTCGATTTTTGTGATGCTCGTCAGGGGGGCGGAGCCTATGGAAAAACGCCAGCAACGCGGCCTTTTTACGGTTCCTGGCCTTTTGCTGGCCTTTTGCTCACATGTTCTTTCCTGCGTTATCCCCTGATTCTGTGGATAACCGTATTACCGCCTTTGAGTGAGCTGATACCGCTCGCCGCAGCCGAACGACCGAGCGCAGCGAGTCAGTGAGCGAGGAAGCGGAAGAGCGCCCAATACGCAAACCGCCTCTCCCCGCGCGTTGGCCGATTCATTAATGCAGCTGGCACGACAGGTTTCCCGACTGGAAAGCGGGCAGTGAGCGCAACGCAATTAATGTGAGTTAGCTCACTCATTAGGCACCCCAGGCTTTACACTTTATGCTTCCGGCTCGTATGTTGTGTGGAATTGTGAGCGGATAACAATTTCACACAGGAAACAGCTATGACCATGATTACGAATTCCCTTGTATCTCTACACACAGGCTCAAATCAATAAGAAGAACGGTTCGTCTTTTTCGTTTATATCTTGCATCGTCCCAAAGCTATTGGCGGGATATTCTGTTTGCAGTTGGCTGACTTGAAGTAATCTCTGCAGATCTTTCGACACTGAAATACGTCGAGCCTGCTCCGCTTGGAAGCGGCGAGGAGCCTCGTCCTGTCACAACTACCAACATGGAGTACGATAAGGGCCAGTTCCGCCAGCTCATTAAGAGCCAGTTCATGGGCGTTGGCATGATGGCCGTCATGCATCTGTACTTCAAGTACACCAACGCTCTTCTGATCCAGTCGATCATCCGCTGAAGGCGCTTTCGAATCTGGTTAAGATCCACGTCTTCGGGAAGCCAGCGACTGGTGACCTCCAGCGTCCCTTTAAGGCTGCCAACAGCTTTCTCAGCCAGGGCCAGCCCAAGACCGACAAGGCCTCCCTCCAGAACGCCGAGAAGAACTGGAGGGGTGGTGTCAAGGAGGAGTAAGCTCCTTATTGAAGTCGGAGGACGGAGCGGTGTCAAGAGGATATTCTTCGACTCTGTATTATAGATAAGATGATGAGGAATTGGAGGTAGCATAGCTTCATTTGGATTTGCTTTCCAGGCTGAGACTCTAGCTTGGAGCATAGAGGGTCCTTTGGCTTTCAATATTCTCAAGTATCTCGAGTTTGAACTTATTCCCTGTGAACCTTTTATTCACCAATGAGCATTGGAATGAACATGAATCTGAGGACTGCAATCGCCATGAGGTTTTCGAAATACATCCGGATGTCGAAGGCTTGGGGCACCTGCGTTGGTTGAATTTAGAACGTGGCACTATTGATCATCCGATAGCTCTGCAAAGGGCGTTGCACAATGCAAGTCAAACGTTGCTAGCAGTTCCAGGTGGAATGTTATGATGAGCATTGTATTAAATCAGGAGATATAGCATGATCTCTAGTTAGCTCACCACAAAAGTCAGACGGCGTAACCAAAAGTCACACAACACAAGCTGTAAGGATTTCGGCACGGCTACGGAAGACGGAGAAGCCACCTTCAGTGGACTCGAGTACCATTTAATTCTATTTGTGTTTGATCGAGACCTAATACAGCCCCTACAACGACCATCAAAGTCGTATAGCTACCAGTGAGGAAGTGGACTCAAATCGACTTCAGCAACATCTCCTGGATAAACTTTAAGCCTAAACTATACAGAATAAGATAGGTGGAGAGCTTATACCGAGCTCCCAAATCTGTCCAGATCATGGTTGACCGGTGCCTGGATCTTCCTATAGAATCATCCTTATTCGTTGACCTAGCTGATTCTGGAGTGACCCAGAGGGTCATGACTTGAGCCTAAAATCCGCCGCCTCCACCATTTGTAGAAAAATGTGACGAACTCGTGAGCTCTGTACAGTGACCGGTGACTCTTTCTGGCATGCGGAGAGACGGACGGACGCAGAGAGAAGGGCTGAGTAATAAGCCACTGGCCAGACAGCTCTGGCGGCTCTGAGGTGCAGTGGATGATTATTAATCCGGGACCGGCCGCCCCTCCGCCCCGAAGTGGAAAGGCTGGTGTGCCCCTCGTTGACCAAGAATCTATTGCATCATCGGAGAATATGGAGCTTCATCGAATCACCGGCAGTAAGCGAAGGAGAATGTGAAGCCAGGGGTGTATAGCCGTCGGCGAAATAGCATGCCATTAACCTAGGTACAGAAGTCCAATTGCTTCCGATCTGGTAAAAGATTCACGAGATAGTACCTTCTCCGAAGTAGGTAGAGCGAGTACCCGGCGCGTAAGCTCCCTAATTGGCCCATCCGGCATCTGTAGGGCGTCCAAATATCGTGCCTCTCCTGCTTTGCCCGGTGTATGAAACCGGAAAGGCCGCTCAGGAGCTGGCCAGCGGCGCAGACCGGGAACACAAGCTGGCAGTCGACCCATCCGGTGCTCTGCACTCGACCTGCTGAGGTCCCTCAGTCCCTGGTAGGCAGCTTTGCCCCGTCTGTCCGCCCGGTGTGTCGGCGGGGTTGACAAGGTCGTTGCGTCAGTCCAACATTTGTTGCCATATTTTCCTGCTCTCCCCACCAGCTGCTCTTTTCTTTTCTCTTTCTTTTCCCATCTTCAGTATATTCATCTTCCCATCCAAGAACCTTTATTTCCCCTAAGTAAGTACTTTGCTACATCCATACTCCATCCTTCCCATCCCTTATTCCTTTGAACCTTTCAGTTCGAGCTTTCCCACTTCATCGCAGCTTGACTAACAGCTACCCCGCTTGAGCAGACATCACCATGGTGAGCAAGGGCGAGGAGGATAACATGGCCATCATCAAGGAGTTCATGCGCTTCAAGGTGCACATGGAGGGCTCCGTGAACGGCCACGAGTTCGAGATCGAGGGCGAGGGCGAGGGCCGCCCCTACGAGGGCACCCAGACCGCCAAGCTGAAGGTGACCAAGGGTGGCCCCCTGCCCTTCGCCTGGGACATCCTGTCCCCTCAGTTCATGTACGGCTCCAAGGCCTACGTGAAGCACCCCGCCGACATCCCCGACTACTTGAAGCTGTCCTTCCCCGAGGGCTTCAAGTGGGAGCGCGTGATGAACTTCGAGGACGGCGGCGTGGTGACCGTGACCCAGGACTCCTCCCTGCAGGACGGCGAGTTCATCTACAAGGTGAAGCTGCGCGGCACCAACTTCCCCTCCGACGGCCCCGTAATGCAGAAGAAGACCATGGGCTGGGAGGCCTCCTCCGAGCGGATGTACCCCGAGGACGGCGCCCTGAAGGGCGAGATCAAGCAGAGGCTGAAGCTGAAGGACGGCGGCCACTACGACGCTGAGGTCAAGACCACCTACAAGGCCAAGAAGCCCGTGCAGCTGCCCGGCGCCTACAACGTCAACATCAAGTTGGACATCACCTCCCACAACGAGGACTACACCATCGTGGAACAGTACGAACGCGCCGAGGGCCGCCACTCCACCGGCGGCATGGACGAGCTGTACAAGTAACCATGACCGAGATCGGCGAGCAGCCGTGGGGGCGGGAGTTCGCCCTGCGCGACCCGGCCGGCAACTGCGTGCACTTCGTGGCCGAGGAGCAGGACTGACCGACGCCGACCAACACCGCCGGTCCGACGGCGGCCCACGGGTCCCAGGAGCTTGAGATCCACTTAACGTTACTGAAATCATCAAACAGCTTGACGAATCTGGATATAAGATCGTTGGTGTCGATGTCAGCTCCGGAGTTGAGACAAATGGTGTTCAGGATCTCGATAAGATACGTTCATTTGTCCAAGCAGCAAAGAGTGCCTTCTAGTGATTTAATAGCTCCATGTCAACAAGAATAAAACGCGTTTTCGGGTTTACCTCTTCCAGATACAGCTCATCTGCAATGCATTAATGCATTGACTGCAACCTAGTAACGCCTTNCAGGCTCCGGCGAAGAGAAGAATAGCTTAGCAGAGCTATTTTCATTTTCGGGAGACGAGATCAAGCAGATCAACGGTCGTCAAGAGACCTACGAGACTGAGGAATCCGCTCTTGGCTCCACGCGACTATATATTTGTCTCTAATTGTACTTTGACATGCTCCTCTTCTTTACTCTGATAGCTTGACTATGAAAATTCCGTCACCAGCNCCTGGGTTCGCAAAGATAATTGCATGTTTCTTCCTTGAACTCTCAAGCCTACAGGACACACATTCATCGTAGGTATAAACCTCGAAATCANTTCCTACTAAGATGGTATACAATAGTAACCATGCATGGTTGCCTAGTGAATGCTCCGTAACACCCAATACGCCGGCCGAAACTTTTTTACAACTCTCCTATGAGTCGTTTACCCAGAATGCACAGGTACACTTGTTTAGAGGTAATCCTTCTTT

Figure S1: Map and sequence of the construct pAN_Hyg_mCherry. The restriction site SmaI was used for linearization prior to transformation.


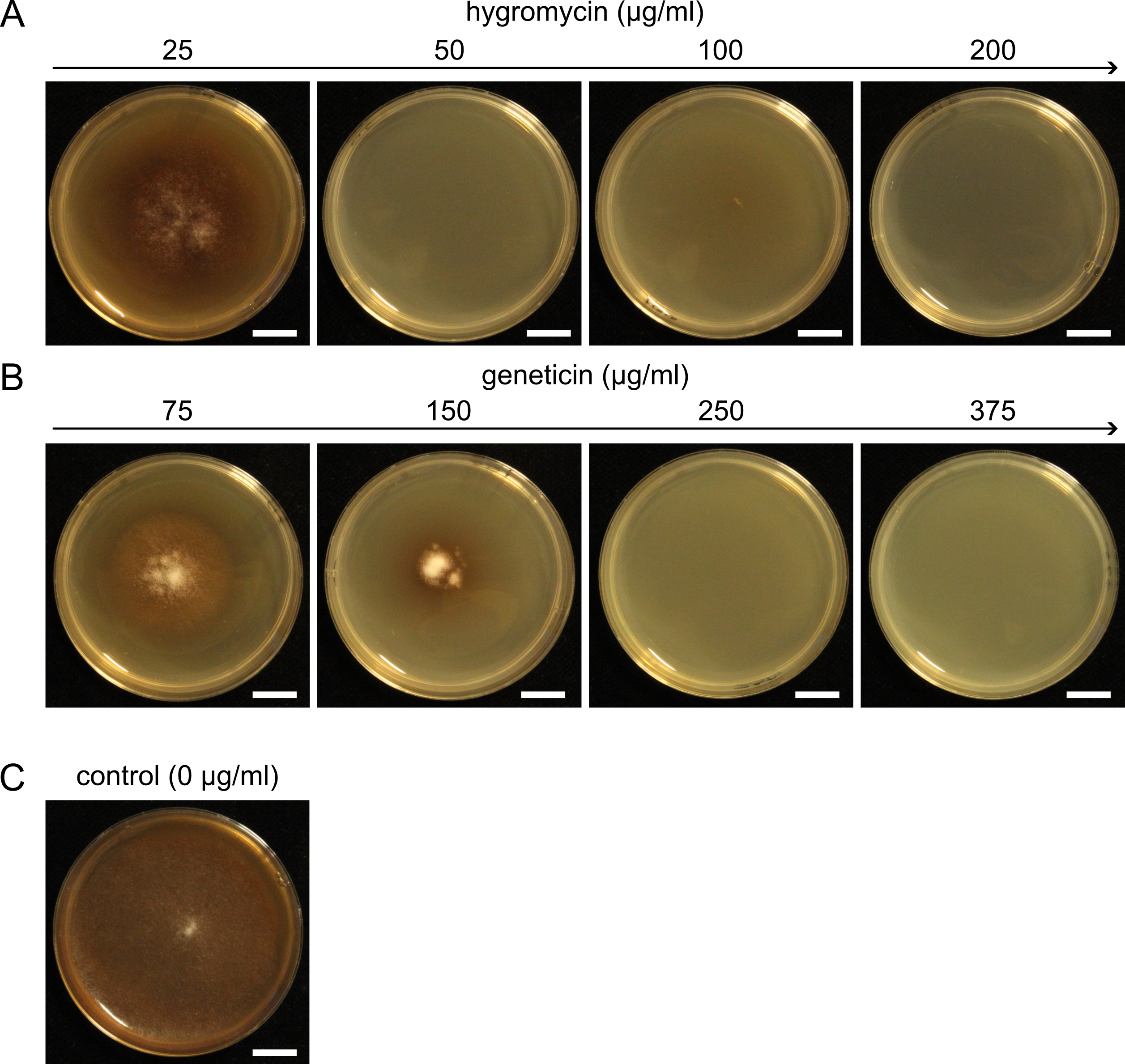


Figure S2: Testing of germinating protoplasts of H. fraxineus NW-FVA 1856 for their sensitivity to hygromycin (**A**) and geneticin (**B**). Mycelium was inoculated on AM_RegS_ containing increasing concentrations of antibiotics. The growth of mycelium was monitored after 21 days of incubation at RT in the dark. Scale bar = 1 cm.

Table S1: Obtained mutants after eleven independent transformations.

| Promoter | Origin | Resistance | Number of transformants |
| --- | --- | --- | --- |
| PtrpC-hph | *Aspergillus nidulans* | Hygromycin | 29 |
|  |  |  | 9 |
|  |  |  | 14 |
| PtrpC-nptII | *Aspergillus nidulans* | Geneticin | 2 |
|  |  |  | 35 |
|  |  |  | 4 |
|  |  |  | 2 |
|  |  |  | 9 |
|  |  |  | 4 |
|  |  |  | 68 |
|  |  |  | 28 |


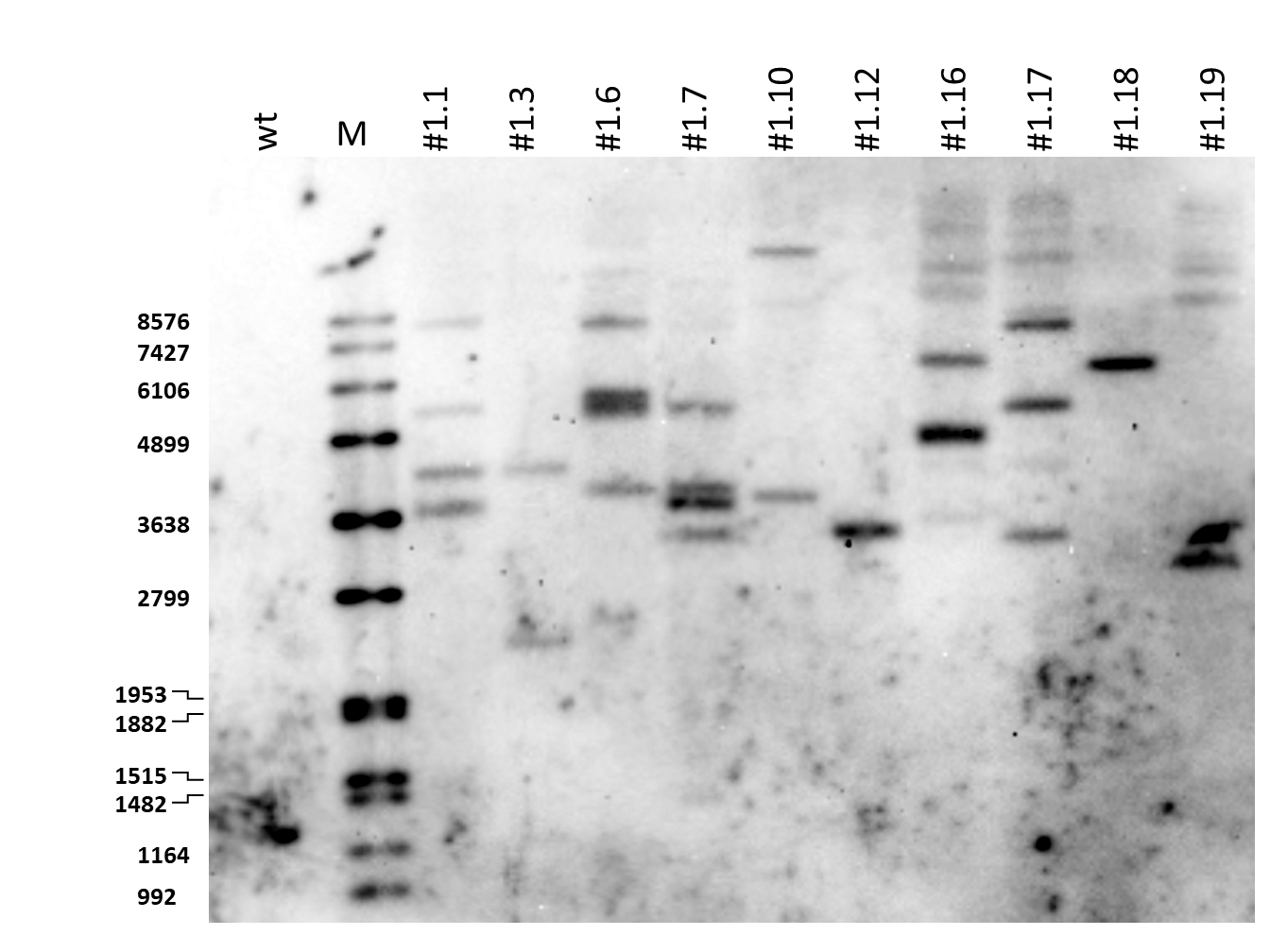


Figure S3: Detection of the number of integrations of pPIGPAPA_Hyg_GFP into the genome of H. fraxineus NW-FVA 1856. Three transformants (#1.12, #1.18 and #1.19) had a single integration, 3 transformants had a double integration (#1.3, #1.10, #1.16) and in 4 transformants, the construct was integrated 3 or 4 times (#1.1, #1.6, #1.7, #1.17). wt= wild type, M=DNA Molecular Weight Marker VII (Roche Penzberg, Germany). The sizes of the marker bands are shown in bp on the left.
